# MicroRNA-98 and microRNA-214 post-transcriptionally regulate enhancer of zeste homolog 2 and inhibit migration and invasion in human esophageal squamous cell carcinoma

**DOI:** 10.1186/1476-4598-11-51

**Published:** 2012-08-06

**Authors:** Sheng-Dong Huang, Yang Yuan, Chong-Wen Zhuang, Bai-Ling Li, De-Jun Gong, Shu-Gang Wang, Zhi-Yong Zeng, He-Zhong Cheng

**Affiliations:** 1 Institute of Cardiothoracic Surgery, Changhai Hospital, 168, Changhai Rd., Shanghai, P. R. China; 2 Department of Cardiothoracic Surgery, Changhai Hospital, Shanghai, P.R. China; 3 Department of Pathology, Changhai Hospital, Second Military Medical University, Shanghai, P.R. China; 4 Department of Cardiothoracic Surgery, Fuzhou General Hospital of Nanjing Command, PLA., Fujian, P.R. China; 5 Department of Pathology, Fuzhou General Hospital of Nanjing Command, PLA, Fujian, P.R. China

**Keywords:** MiR-98, MiR-214, EZH2, ESCC, Migration, Invasion

## Abstract

**Background:**

The enhancer of zeste homolog 2 (EZH2) was found to be overexpressed and associated with tumor metastasis in esophageal squamous cell carcinoma (ESCC). On the other hand, it was reported that miR-26a, miR-98, miR-101, miR-124, miR-138 and miR-214 could inhibit the expression of EZH2 in some tumors. However, the role of miRNAs in the regulation of EZH2 expression in human ESCC has not been documented. The aim of this study was to determine the role of these miRNAs in the regulation of tumor metastasis via EZH2 overexpression in human ESCC.

**Methods and results:**

The expression of these miRNAs and EZH2 mRNA were examined by qPCR and the expression of EZH2 protein was detected by western blot. The role of these miRNAs in migration and invasion was studied in ESCC cell line (Eca109) transfected with miRNA mimics or cotransfected with miRNA mimics and pcDNA-EZH2 plasmid (without the 3’-UTR of EZH2). Through clinical investigation, we found that miR-98 and miR-214 expression was significantly lower in ESCC tissues than in matched normal tissues, and the expression level of miR-98 and miR-214 was inversely correlated to EZH2 protein expression and the clinical features such as pathological grade, tumor stage and lymph node metastasis in ESCC. In Eca109 cells, overexpression of miR-98 and miR-214 significantly inhibited the migration and invasion of ESCC cells, which was reversed by transfection of EZH2.

**Conclusions:**

These findings suggest that decreased expression of miR-98 and miR-214 might promote metastasis of human ESCC by inducing accumulation of EZH2 protein.

## Introduction

Esophageal squamous cell carcinoma (ESCC) is the second most common cancer in China [1]. The recurrence rate of ESCC is extremely high after surgical treatment and the prognosis is usually poor [2,3]. Metastasis is a strong independent prognostic factor for ESCC [4,5]. Therefore, any insight into the mechanisms of ESCC metastasis may provide important clues for the development of clinical diagnostic methods and effective therapeutics [6].

The enhancer of zeste homolog 2 (EZH2, also called histone lysine methyltransferase) is located at chromosome 7q35 and encodes a member of the Polycomb group proteins [7], which regulate gene expression via epigenetic modification of chromatin structure including inducing histone acetylation and methylation [7,8]. Previous studies showed that EZH2 is overexpressed in a broad range of tumors [9,10]. Moreover, increased EZH2 expression could significantly promote tumor cell migration and invasion, and is strongly associated with tumor metastasis and poor clinical prognosis in a variety of human tumors such as breast, prostate, endometrial, gastric, colon, hepatocellular, bladder and oral cancers [11-18]. In the case of human ESCC, He et al. [19] and Tzao et al. [20] independently reported that overexpression of EZH2 is associated with tumor metastasis and poor prognosis of the patients. However, the mechanism underlying EZH2 overexpression in ESCC remains unclear.

In recent years, accumulating data indicate that expression of EZH2 is regulated at the posttranscriptional level by a number of microRNAs (miRNAs). It was reported that miR-26a, miR-98, miR-101, miR-124, miR-138 and miR214 inhibit the expression of EZH2 in nasopharyngeal carcinoma, nasopharyngeal carcinoma, glioblastoma, hepatocellular carcinoma, head and neck squamous cell carcinoma, and neuroblastoma, respectively [21-26]. However, the role of miRNAs in the regulation of EZH2 expression in human ESCC has not been documented. Considering that the expression and function of miRNAs may vary in different types of tumors, here we set out to investigate whether these miRNAs (miR-26a, miR-98, miR-101, miR-124, miR-138 and miR214) regulate tumor metastasis via altering EZH2 expression in human ESCC. Through clinical investigation and cellular experiments using ESCC cell line, we demonstrate that decreased expression of miR-98 and miR-214 induce accumulation of EZH2 protein and might thereby promote the metastasis of human ESCC.

## Results

### EZH2 expression is up-regulated at the post-transcriptional level in ESCC specimens

**Figure 1:**
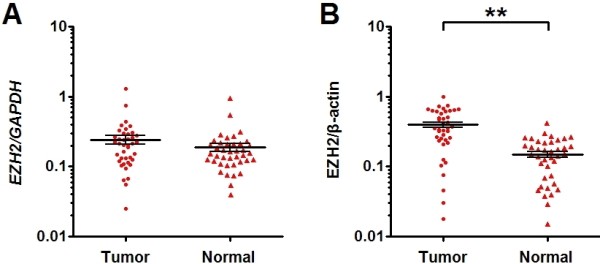
**EZH2 protein but not mRNA expression is increased in ESCC specimens as compared to matched normal tissues.** (**A**, **B**) The expression of EZH2 mRNA and protein in the ESCC specimens and the matched normal tissues was detected by qRT-PCR and western blot and normalized to that of *GAPDH* and β-actin, respectively. Results showed that the expression of EZH2 mRNA (**C**) was comparable between tumor tissue and matched normal tissue, while the expression of EZH2 protein (**D**) was significantly increased in tumor tissue compared with the matched normal tissue. Data are presented as individual samples (n = 40) with the line indicating the mean level; **, *P* < 0.01 by paired t test.

In the present study, we examined EZH2 mRNA and protein expression in ESCC tissues and matched normal esophageal tissues by qRT-PCR and western blot analysis. EZH2 mRNA appeared to be higher in tumor than in the normal tissue in 60% (24/40) of specimens, but overall the difference in EZH2 mRNA expression between ESCC tissues and the matched normal tissues was not statistically significant (Figure 1A). On the other hand, the expression level of EZH2 protein was upregulated in tumor in 92.5% (37/40) of specimens with a total average of 2.6 fold increase than in the matched normal tissues (Figure 1B, Additional file 1: Figure S1). These data suggest that EZH2 expression is up-regulated at the posttranscriptional level in human ESCC.

### MiR-98, miR-101 and miR214 expression is down-regulated in ESCC specimens

**Figure 2:**
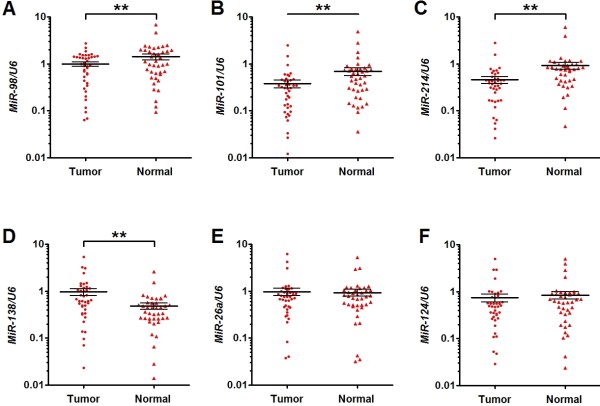
**MiR-98, miR-101 and miR-214 expression is decreased in ESCC specimens as compared to matched normal tissues.** The expression of miRNAs in the ESCC specimens and the matched normal tissues was detected by qRT-PCR and normalized to that of *U6*. Results showed that the expression of miR-98 (**A**), miR-101 (**B**) and miR-214 (**C**) were significantly decreased in tumor tissue compared with the matched normal tissue; while that of miR-138 (**D**) was significantly increased in tumors tissue, and there was no significantly difference in the expression of miR-26a (**E**) and miR-124 (**F**) between the two groups. Data are presented as individual samples (n = 40) with the line indicating the mean level; **, *P* < 0.01 by paired t test.

MiR-26a, miR-98, miR-101, miR-124,miR-138 and miR-214 were reported to be decreased in some human tumors and posttranscriptionally regulate the expression of EZH2 [21-26]. In the present study, we first examined the expression levels of MiR-26a, miR-98, miR-101, miR-124, miR-138 and miR214 in clinical samples of ESCC and matched normal tissues using qPCR. Expression of miR-98 and miR-101 was found to be downregulated in tumor tissues compared with the matched normal tissues in 67.5% (27/40) of samples. In 77.5% (31/40) of samples, miR-214 was found to be downregulated. In 42.5% (17/40) of samples, all the three miRNAs were downregulated in tumor versus normal tissues. Figure 2A-C shows the mean expression levels of miR-98, miR-101 and miR-214, which were significantly lower in tumor tissues than in matched normal tissues. On the other hand, expression of miR-138 was significantly higher in tumor tissues than in matched normal tissues (Figure 2D) and the expression levels of miR-26a and miR-124 were not significantly different between tumor and matched normal tissues (Figure 2E and F).

**Figure 3:**
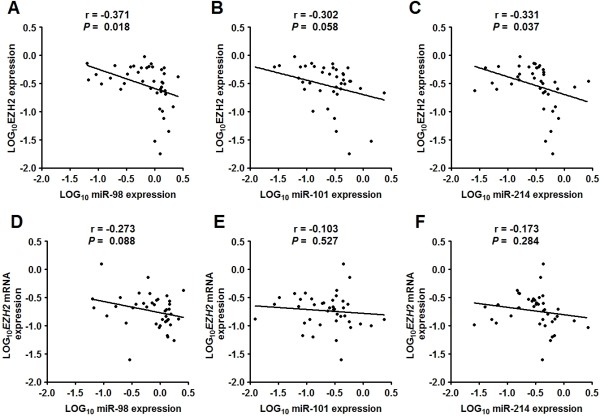
**MiR-98 and miR-214 expression is inversely correlated with that of EZH2 protein.** Dot plots represent log_10_EZH2 protein (mRNA) relative expression level against log_10_miRNA relative expression level. The lines represent approximated curves. The correlation coefficient (r) and the *P* value indicate the statistical significance of the negative correlation between the x and y variables. Results showed that the expression of miR-98 was inversely correlated with EZH2 protein (**A**) but not mRNA expression (**B**) in tumor cells. Similarly, the expression of miR-214 was inversely correlated with EZH2 protein (**C**) but not mRNA expression (**D**). There was no significant association between the expression of miR-101 and EZH2 protein (**E**) and mRNA (**F**).

We further investigated the relationship of the expression level of miRNAs (miR-98, miR-101 and miR214) with EZH2 expression. It was found that 75% (30/40), 62.5% (25/40), and 85% (34/40) of samples showed downregulation of miRNAs (i.e. miR-98, miR-101 and miR214) with upregulation of EZH2 protein in tumor versus normal tissues, respectively. These results suggest that downregulation of these miRNAs might contribute to the accumulation of EZH2 protein in tumors. Moreover, the expression of miR-98 (or miR-214) was inversely correlated with EZH2 protein but not mRNA expression in tumor tissues (Figure 3A-D). On the other hand, there appeared to be no significant association between the expression of miR-101 and EZH2 mRNA and protein (Figure 3E-F). These data suggest that miR-98 and miR-214 may play an important role in regulating the expression of EZH2 protein in human ESCC.

### MiR-98, miR-101 and miR214 are correlated with pathological grade, tumor stage and lymph node metastasis

We further analyzed the relationship of miR-98, miR-101 and miR214 expression with the clinical features including age, gender, pathological grade, tumor location, tumor stage and lymph node metastasis in ESCC. For each miRNA, patients were divided into two groups, high expression (meaning higher than the mean level) and low expression (meaning lower than the mean level). It was found that the expression of miR-98 (or miR-214) was significantly correlated with pathological grade, tumor stage and lymph node metastasis (Table 1). MiR-101 expression was also significantly correlated with tumor stage and lymph node metastasis (Table 1). These results suggest that the miR-98, miR-101 and miR214 might be involved in metastasis of ESCC.

**Table 1:** Relationship between the expression of miRNAs (miR-98, miR-101 and miR-214) and clinical features

**Clinical features**	**MiR-98**			**MiR-101**			**MiR-214**		
	**High**	**Low**	***P***	**High**	**Low**	***P***	**High**	**Low**	***P***
**Total**	20	20		13	27		12	28	
**Age (year)**									
<60	7	9	0.519	7	9	0.215	4	12	0.573
≥60	13	11		6	18		8	16	
**Gender**									
Male	16	12	0.168	7	21	0.122	8	20	0.763
Female	4	8		6	6		4	8	
**Pathological grading**									
Well	11	2	0.004	7	6	0.135	8	5	0.010
Moderately	6	7		3	10		2	11	
Poorly	3	11		3	11		2	12	
**Tumor location**									
Upper1/3-middle1/3	15	12	0.311	8	19	0.576	9	18	0.507
Lower1/3	5	8		5	8		3	10	
**Tumor stage**									
T1/T2	12	3	0.003	9	6	0.004	8	7	0.013
T3/T4	8	17		4	21		4	21	
**Lymph node metastasis**									
Negative	13	4	0.004	9	8	0.018	8	9	0.043
Positive	7	16		4	19		4	19	

### MiR-98, miR-101 and miR-214 posttranscriptionally down-regulates EZH2 expression in ESCC cell line

**Figure 4:**
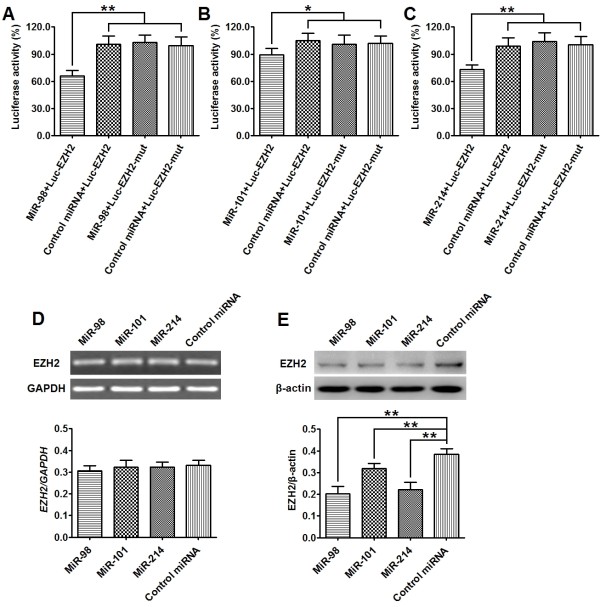
**MiR-98, miR-101 and miR-214 posttranscriptionally down-regulates EZH2 expression in ESCC cell line. (A-C)**1 × 10^6^ Eca109 cells were cotransfected with 50 pmol of miRNAs (or control miRNA) and 1 μg of Luc-EZH2 (or matched Luc-EZH2-mut) plasmid, respectively. Luciferase reporter assay were performed at 48 hr posttransfection. Results showed that cells transfected with miR-98 + Luc-EZH2 (**A**), miR-101 Luc-EZH2 (**B**), miR-214 Luc-EZH2 (**C**) exhibited a significant decrease of reporter activity in comparison with those cotransfected with control microRNA + Luc-EZH2 plasmid. However, the reporter activity of cells cotransfected with miRNAs and Luc-EZH2-mut plasmid showed no significant difference with that of cells cotransfected with control miRNA and Luc-EZH2-mut plasmid. (**D**, **E**) 1 × 10^6^ Eca109 cells were transfected with 50 pmol of miRNAs (or control microRNA), respectively. The expression level of EZH2 mRNA and protein was detected by qPT-PCR an Western Blot at 48 hr posttransfection and normalized to that of GAPDH and β-actin, respectively. Results showed that the level of EZH2 protein (**D**) was significantly decreased in cells transfected with miRNAs (including miR-98, miR-101 and miR-214) as compared to the cells transfected with control microRNA, while the expression level of *EZH2* mRNA (**E**) exhibited no significantly difference between cells transfected with miRNAs and those transfected with control microRNA. Data represent mean ± SEM from 4 independent experiments; *, *P* < 0.05, **, *P* < 0.01 by t test.

To determine whether the 3'-UTR of *EZH2* mRNA is a functional target of miR-98, miR-101 and miR-214 in ESCC cells, we measured the luciferase activity in cells cotransfected with these miRNAs (or control miRNA) and Luc-EZH2 plasmid (or Luc-EZH2-mut plasmid) in ESCC cell line (Eca109). qPCR analysis confirmed that Eca109 cells transfected with miRNA mimics (i.e miR-98, miR-101 or miR-214) exhibited significantly higher mature miRNA level than those treated with controls 48 hr posttransfection (Additional file 2: Figure S2). As shown in Figure 4A-C, cells cotransfected with miRNAs (ie, miR-98, miR-101 or miR-214) and Luc-EZH2 plasmid showed a significant decrease of reporter activity in comparison with those cotransfected with control microRNA and Luc-EZH2 plasmid. However, the reporter activity were comparable between cells cotransfected with miRNAs (miR-98, miR-101 ormiR-214) and Luc-EZH2-mut plasmid and cells cotransfected with control microRNA and Luc-EZH2-mut plasmid (Figure 4A-C). We further detected the expression of EZH2 protein and mRNA by western blot and qRT-PCR in Eca109 cells transfected with miRNAs (miR-98, miR-101 or miR-214). Expression of EZH2 protein was found to be significantly decreased in cells transfected with miRNAs (miR-98. miR-101 or miR-214) as compared to cells treated with control microRNA (Figure 4D). However, *EZH2* mRNA expression was not significantly different between the 2 groups (Figure 4E). These results indicate that the 3'-UTR of *EZH2* mRNA is a functional target of miR-98, miR-101 and miR-214 in ESCC cells.

### **MiR-98, miR-101 and miR-214 inhibit the migration and** invasion **of ESCC cell line**

**Figure 5:**
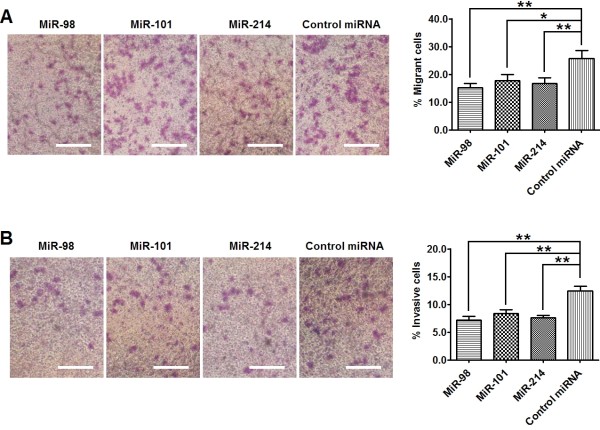
**MiR-98, miR-101 and miR-214 inhibits the migration and invasion of ESCC cells.** Eca109 cells were transfected with 50 pmol of miRNAs (or control microRNA). Migration and invasion of cells were analyzed at 72 hr post-transfection. (**A**) Transwell assay. Photographs represented the cells travelled through the micropore membrane and histogram showed the percentage of migrant cells. (**B**) Matrigel-coated transwell assay. Photographs represented the cells invaded through the matrigel and histogram showed the percentage of invasive cells. Bars = 100 μm. Data represent mean ± SEM from 4 independent experiments; *, *P* < 0.05; **, *P* < 0.01 by t test.

To investigate the role of miR-98, miR-101 and miR-214 in ESCC metastasis, we detected the migrant and invasive capacity of Eca109 cells transfected with miRNA mimics or control miRNA. Through transwell assay, we found that the percentage of cells travelled through the micropore membrane was significantly decreased in cells transfected with miRNAs (miR-98, miR-101 or miR-214) as compared to those cells transfected with control miRNA (Figure 5A). In addition, using matrigel-coated transwell assay, we found that the percentages of cells that invaded through the matrigel in miRNAs (miR-98, miR-101 or miR-214) transfected cells were significantly lower than those in the control groups (Figure 5 B). These results indicate that miR-98, miR-101 and miR-214 could inhibit the migration and invasion of ESCC cells.

### Overexpression **of EZH2 reverses the inhibition of migration and invasion of ESCC cells by miR-98 and miR-214**

**Figure 6:**
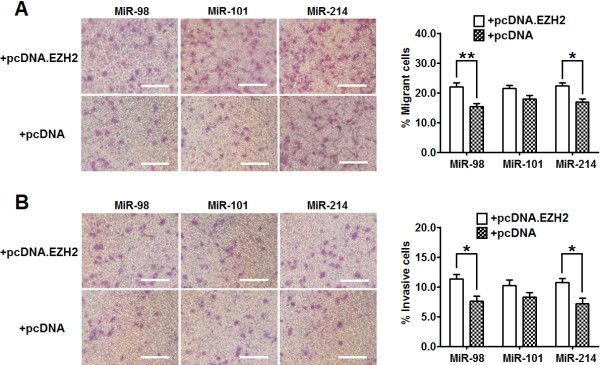
**Re-expressing EZH2 significantly attenuates the effect of miR-98 and miR-214 on the inhibition of ESCC cell migration and invasion.** Eca109 cells were co-transfected with 50 pmol of miRNAs and 1 μg of pcDNA-EZH2 (or empty pcDNA3.1) plasmid. Migration and invasion of cells were analyzed at 72 hr post-transfection. (**A**) Transwell assay. Photographs represented the cells travelled through the micropore membrane and histogram showed the percentage of migrant cells. (**B**) Matrigel-coated transwell assay. Photographs represented the cells invaded through the matrigel and histogram showed the percentage of invasive cells. Bars = 100 μm. Data represent mean ± SEM from 4 independent experiments; *, *P* < 0.05; **, *P* < 0.01 by t test.

To investigate the functional connection between miRNAs (miR-98, miR-101 and miR-214) and EZH2 in the regulation of ESCC metastasis, we further evaluated the migration and invasion capacity of cells cotransfected with these miRNAs and pcDNA-EZH2 (or empty pcDNA) plasmid. Notably, the pcDNA-EZH2 plasmid was designed to carry the open reading frame of human *EZH2* without 3'-UTR. As shown in Additional file 3: Figure S3, EZH2 protein expression was significantly higher in pcDNA-EZH2 transfected cells than in pcDNA transfected cells. Compared with cells cotransfected with miR-98 (or miR-214) and empty cDNA plasmid, a significantly higher percentage of cells cotransfected with miR-98 (or miR-214) and pcDNA-EZH2 plasmid travelled through the micropore or invaded through the matrigel (Figure 6A and B). However, the percentage of cells that travelled through the micropore or invaded through the matrigel were comparable between cells cotransfected with miR-101 and pcDNA-EZH2 plasmid and cells cotransfected with miR-101 and pcDNA plasmid (Figure 6A and B). These results show that overexpression of EZH2 could reverse the inhibitory effect of miR-98 and miR-214 on cell migration and invasion.

## Discussion

EZH2 has been identified as a transcriptional repressor and is implicated in the aggressiveness and metastasis of many types of human cancers including ESCC [19,20]. In recent years, it was reported that some miRNAs could regulate EZH2 expression at the post-transcriptional level in several types of tumors [21-26]. In the present study, we show that miR-98 and miR-214 expression in ESCC tissue are inversely correlated with the clinical features such as pathological grade, tumor stage and lymph node metastasis. In Eca109 cells, overexpressing miR-98 and miR-214 was found to significantly suppress cell migration and invasion through inhibition of EZH2 expression.

Previous studies showed that the expression of EZH2 is regulated at the transcriptional and posttranscriptional levels [21-27]. Tang et al. [27] reported that p53 could inhibit the transcription of EZH2 by binding to the promoter of EZH2 in prostate cancer. On the other hand, mounting evidence indicate that some miRNAs could effectively repress the expression of EZH2 in tumors such as breast, prostate, endometrial, gastric, colon, hepatocellular, bladder and oral cancers [21-26]. In the present study, we detected EZH2 protein and mRNA expression in ESCC tumor tissues and matched normal tissues by western blot and qRT-PCR. It was found that the expression level of EZH2 protein was significantly higher in tumor tissues than in matched normal tissues, despite that *EZH2 mRNA* expression was comparable between the two groups. These results suggest that EZH2 expression is upregulated in human ESCC mainly at the posttranscriptional level.

MiRNAs are evolutionarily conserved small noncoding RNAs (21–25 nucleotides) that regulate gene expression through modulation of translation efficiency or degradation of mRNAs [28,29]. It was reported that miR-26a, miR-98, miR-101, miR-124, miR-138 and miR-214 were involved in the regulation of EZH2 expression in some human tumors such as nasopharyngeal carcinoma, nasopharyngeal carcinoma, glioblastoma, hepatocellular carcinoma, head and neck squamous cell carcinoma, and neuroblastoma [21-26]. In the present study, we compared the expression level of these miRNAs in ESCC tissues and matched normal tissues by qPCR. We found that expression levels of miR-98, miR-101 and miR-214 were significantly lower in tumor than in normal tissues. On the other hand, miR-138 expression was significantly higher in tumor than in normal tissues and miR-26a and miR-124 expression was comparable between the two types of tissues. Using luciferase assay and western blot, we further demonstrated that miR-98, miR-101 and miR-214 could target the 3’-URT of EZH2 and suppress EZH2 expression in ESCC cells. Combining these findings, we propose that miR-98, miR-101 and miR-214 regulate the accumulation of EZH2 protein in ESCC.

MiR-98 belongs to the mature let-7 family of miRNAs [30] and was initially found to be down-regulated in leukemia cell lines [31]. Subsequent studies showed that the expression of miR-98 were also significantly decreased in solid tumors such as nasopharyngeal carcinoma, head and neck squamous cell carcinoma [22,32]. Therefore, miR-98 is wildly regarded as a tumor suppressor gene. On the other hand, the expression and function of miR-214 appears to be cell type- and disease-specific. It was reported that miR-214 was down-regulated in breast and cervical cancer and acted as a tumor suppressor gene in these tumors since its overexpression inhibits cell proliferation and invasion [26,33]. By contrast, other studies showed that miR-214 was over-expressed in pancreatic and ovarian cancers and its overexpression promotes cell survival and chemotherapy resistance [34,35]. In the present study, we found that miR-98 and miR-214 expression were both inversely correlated with EZH2 protein expression in human ESCC, and that down-regulation of miR-98 and miR-214 expression was significantly correlated with pathological grading, tumor stage and lymph node metastasis. Moreover, overexpression of miR-98 or miR-214 could significantly inhibit ESCC cell migration and invasion, which was reversed by over-expressing EZH2. These findings suggest that miR-98 and miR-214 may play an important role in inhibiting the metastasis of ESCCs by targeting EZH2.

MiR-101 was reported to be down-regulated in human colon cancer, nasopharyngeal carcinoma, neuroblastoma and prostate cancer, and could repress the proliferation, invasion and metastasis of tumor cells [22,23,36,37]. In the present study, we found that miR-101 expression was down-regulated in primary ESCC tumor tissues and was significantly correlated with the tumor stage and lymph node metastasis. Although in Eca109 cells, over expression of miR-101 was found to suppress EZH2 expression, we did not detect significant correlation between the expression of miR-101 and EZH2 in clinical samples of ESSC tumor tissues and we found that miR-101-induced inhibition of Eca109 migration and invasion was not reversed by overexpressing EZH2. The discrepancies might be attributable to the regulation of EZH2 expression by multiple miRNAs, amongst which miR-101 only plays a minor role. Further studies are needed to test whether miR-101 might inhibit ESCC metastasis via an EZH2-independent signal pathway.

In summary, we have demonstrated that the expression of miR-98 and miR-214 was significantly lower in ESCC tissues than in matched normal tissues and that down-regulation of miR-98 and miR-214 was correlated with the up-regulated EZH2 protein expression, poor pathological grade, advanced tumor stage and lymph node metastasis in ESCC. In Eca109 cells, miR-98 and miR-214 overexpression significantly inhibited cell migration and invasion by repressing EZH2 protein expression. We propose that miR-98 and miR-214 are tumor suppressor genes in ESCC. It would be interesting to test whether these miRNAs act synergistically to regulate ESCC cell migration and invasion. Further experiments in animal models are warranted to establish the role of these miRNAs and EZH2 in regulating ESCC metastasis.

## Material and method

### ESCC specimens

A total of forty primary ESCC patients that underwent esophagectomy were enrolled in this study. Tumor specimens and paired normal esophageal tissue specimens taken from a site distant from the cancerous lesion were obtained from the consenting patients, as approved by the Medical Ethics Committee of Changhai Hospital. None of the patients received radiotherapy or chemotherapy before surgery. Clinical and pathological data including age, gender, pathological grading, tumor location, tumor stage and lymph node metastasis were acquired from the medical records.

### Cell culture

Human ESCC cell line Eca109 was purchased from the Shanghai Institute of Biochemistry and Cell Biology (Shanghai, China). Cells were maintained in RPMI1640 (Invitrogen) supplemented with 10% fetal bovine serum (Invitrogen), 100 U/ml penicillin and 100 μg/ml streptomycin, within a humidified atmosphere containing 5% CO_2_ at 37°C.

### Cell transfection

Cells were cultured to 1 × 10^6^/ well in 6-well cell culture plate and were then transfected with 50 pmol of miRNA double-stranded mimics (or control miRNA) using Lipofectamine 2000 (Invitrogen) according to the manufacturer’s protocol Transfection efficiency was optimized using 6-carboxyfluorescein-labeled microRNA at approximately 80% in Eca109 cells.

The sequences of miR-98 were:

Sense: 5^′^- UGAGGUAGUAAGUUGUAUUGUU −3^′^,

Anti-sense: 5^′^- AACAAUACAACUUACUACCUCA −3^′^,

The sequences of miR-101 were:

Sense: 5^′^- UACAGUACUGUGAUAACUGAA −3^′^,

Anti-sense: 5^′^- UUCAGUUAUCACAGUACUGUA −3^′^,

The sequences of miR-214 were:

Sense: 5^′^- ACAGCAGGCACAGACAGGCAGU −3^′^,

Anti-sense: 5^′^- ACUGCCUGUCUGUGCCUGCUGU −3^′^,

A scrambled microRNA with no homology to any known human microRNA was used as negative control:

Sense: 5^′^-GUUGAACUGUUAAGAACCACUGG-3^′^,

Anti-sense: 5^′^-CCAGUGGUUCUUAACAGUUCAAC-3^′^,

All microRNA mimics were synthesized by Genephama Biotech (Shanghai, China).

### Quantitative reverse transcription polymerase chain reaction (qRT-PCR)

Total RNA was extracted from 100 mg tissues or 1 × 10^5^ cells using the RNeasy RNA Mini Kit (Qiagen). First strand cDNA was synthesized using POWERSCRIPT reverse transcriptase (Clontech). The following gene-specific primer pairs were used for quantitative PCR:

*EZH2*: Forward, 5^′^- TTACTTGTGGAGCCGCTGAC -3^′^;

Reverse, 5^′^- TCAGATGGTGCCAGCAATAG-3^′^.

*GAPDH*: Forward, 5^′^- GCTGAGTATGTCGTGGAGTC -3^′^;

Reverse, 5^′^- AGTTGGTGGTGCAGGATGC -3^′^.

PCR was performed using a Fast Start Master SYBR Green Kit (Roche) on a LightCycler (Roche). The expression level of target gene mRNA was analyzed using RealQuant software (Roche) and normalized to that of *GAPDH* mRNA.

### Cell lysis and western blot

Cellular proteins were prepared using cell lysis buffer (50 mM Tris–HCl, pH 8.0, 1% NP-40, 2 mM EDTA, 10 mM NaCl, 2 mg/ml aprotinin, 5 mg/ml leupeptin, 2 mg/ml pepstatin, 1 mM DTT, 0.1% SDS and 1 mM phenylmethylsulfonyl fluoride). Equal amounts of protein (50 μg) were separated by 10% SDS PAGE and then transferred to nitrocellulose membranes (NY, USA) by electroblotting. The membranes were blocked with 5% BSA in TBST (10 mM Tris–HCl, pH 8.0, 150 mM NaCl, and 0.05% Tween 20) for 1 hr, and then incubated with mouse anti-human EZH2 antibody (Santa Cruz) overnight at 4°C before subsequent incubation with horseradish peroxidase-conjugated goat anti-mouse antibody (BD) for 1 hr at 37°C. Protein was visualized using enhanced chemiluminescence reagent (Santa Cruz). The expression level of EZH2 protein was analyzed using LabWork 4.0 program (UVP) and normalized to that of β-actin protein.

### Luciferase reporter assay

The full-length 3^′^-UTR of *EZH2* mRNA was amplified by PCR (Forward: 5^′^-CTAGTCATCTGCTACCTCCTCC-3^′^; Reverse: 5^′^-AGCTTACAAGTTCAAGTATTCTTTATTC-3^′^). Mutant *EZH2* 3^′^-UTR, which carried a substitution of four nucleotides (AGGU to UCCA for miR-98, UACU to AUGA for miR-101, and CAGC to GUCG for miR-214) within the core binding sites of *EZH2* 3^′^-UTR, was obtained using overlapping extension PCR. Normal (or mutant) *EZH2* 3^′^-UTR was cloned into the SacI-HindIII site of the pMIR-REPORT luciferase vector (Biosystems) and named as Luc-EZH2 (or Luc-EZH2-mut). Then, 1 × 10^6^ cells were cotransfected with 50 pmol of miRNAs (or control miRNA), 1 μg of Luc-EZH2 (or matched Luc-EZH2-mut) plasmid, and 1 μg of pMIR-REPORT β-Gal vector using Lipofectamine 2000. The Luciferase activity was examined at 48 hr posttransfection using the luciferase assay kit (Clontech) and normalized to β-galactosidase activity.

### Transwell assay

Cell migration and invasion were determined using a transwell (Costar) with a pore size of 0.8 μm. 1 × 10^3^ cells were seeded in serum-free medium in the upper chamber (normal chamber for migration assay and matrigel-coated chamber for invasion assay). The lower chamber was filled with medium containing 10% FBS. After incubating for 8 hr at 37°C, cells in the upper chamber were carefully removed with a cotton swab and the cells that had traversed to reverse face of the membrane were fixed in methanol, stained with Giemsa, and counted.

### Statistical analysis

Statistical significance was tested using SPSS15.0 software. For comparison of clinical features (except for pathological grading) between high and low miRNA expression groups, chi-square test was performed. The correlation between the expression of miRNA and pathological grade was analyzed by Cochran-Mantel-Haenszel Statistics. The correlation between the expression of miRNA and EZH2 was analyzed using Pearson’s correlation analysis. Other data are presented as mean ± SEM, using student *t* tests for 2-group comparison. A *P* value less than 0.05 is considered as statistically significant.

## Abbreviations

miR: microRNA; ESCC: Esophageal squamous cell carcinoma; EZH2: Enhancer of zeste homolog 2; 3’-UTR: 3’ untranslated region.

## Competing interests

The authors declare that they have no competing interests.

## Authors' contributions

SDH and ZYZ designed research and analyzed data. YY, HZC, BLL, DJG, CWZ and SGW carried out molecular biology studies. YY and HZC wrote the paper. All authors read and approved the final manuscript.

## Supplementary Material

Additional file 1 Figure S1.The expression level of EZH2 protein in ESCC and paired normal tissues. Click here for file

Additional file 2 Figure S2.The expression levels of miRNAs were significantly increased in Eca109 cells transfected with miRNA mimics. Click here for file

Additional file 3 Figure S3.The expression levels of EZH2 was significantly increased in Eca109 cells transfected with pcDNA.EZH2. Click here for file
