# Supplementary material for: MicroRNA-98 and microRNA-214 post-transcriptionally regulate enhancer of zeste homolog 2 and inhibit migration and invasion in human esophageal squamous cell carcinoma
Source: Mol Cancer. 2012 Aug 6;11:51. doi: 10.1186/1476-4598-11-51 (PMC3496689; doi:10.1186/1476-4598-11-51)
Supplement: Additional file 1 — Figure S1.The expression level of EZH2 protein in ESCC and paired normal tissues. [file 1476-4598-11-51-S1.pdf]

Supplement Fig 1

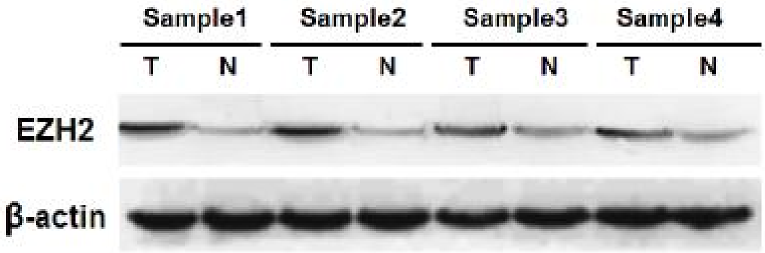

Fig.S1 The expression level of EZH2 protein in ESCC and paired normal tissues.

Total cell protein was extracted from 100mg tissues, and then western blot was performed as described in materials and methods. Graph represents EZH2 protein expression in the representative tumor and paired normal tissues. It was showed that EZH2 protein was highly expressed in ESCC tissues than in the paired normal tissues.
