# Supplementary material for: MicroRNA-98 and microRNA-214 post-transcriptionally regulate enhancer of zeste homolog 2 and inhibit migration and invasion in human esophageal squamous cell carcinoma
Source: Mol Cancer. 2012 Aug 6;11:51. doi: 10.1186/1476-4598-11-51 (PMC3496689; doi:10.1186/1476-4598-11-51)
Supplement: Additional file 2 — Figure S2.The expression levels of miRNAs were significantly increased in Eca109 cells transfected with miRNA mimics. [file 1476-4598-11-51-S2.pdf]

Supplement Fig 2

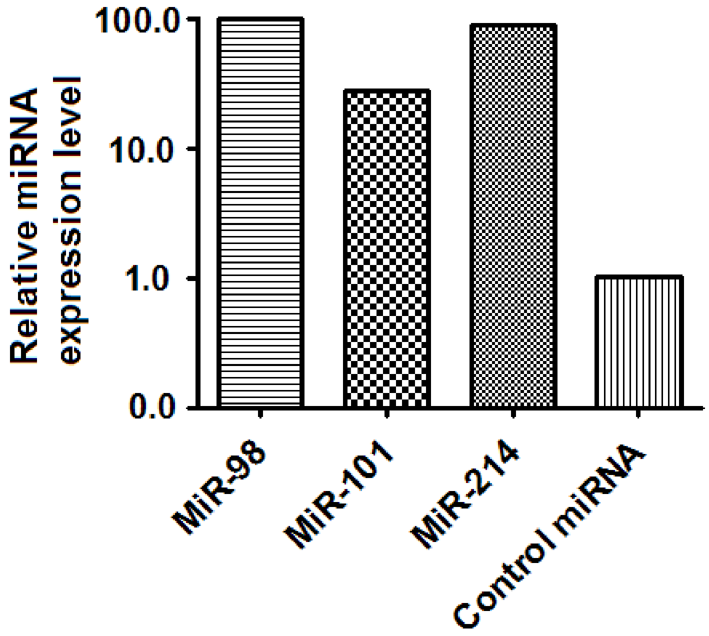

Fig. S2 The expression levels of miRNAs were significantly increased in Eca109 cells transfected with miRNA mimics.

Total RNA was extracted from 1×10^5 cells using the miRNeasy Mini Kit (QIAGEN). Reverse transcription reactions and real-time PCR reactions were performed using miScript PCR Starter Kit (QIAGEN) and Hs_miRNA miScript Primer Assay system (QIAGEN) according to the manufacturer's protocol. PCR was performed on a LightCycler and analysis was carried out using RealQuant software. Threshold cycle (Ct) values were assigned according to the cycle number at which a fixed fluorescent intensity was achieved. A ΔCt value was calculated by Ct (miRNA) - Ct (U6). Fold induction of mature miRNA expression level in the miRNA mimics (or control miRNA) transfected cells relative to the untreated cells was then calculated as $2^{-\Delta\Delta Ct}$, where ΔΔCt is the difference between ΔCt (microRNA treated cells) and ΔCt (untreated cells). Our data showed that the level of mature miR-98, miR-101, and miR-214 in Eca109 cells transfected with miRNA mimics was about 97-fold, 35-fold, and 83-fold higher than that in cells transfected with control microRNA at 48 hr posttransfection, respectively. **, *P* < 0.01 by t test.
