# Supplementary material for: MicroRNA-98 and microRNA-214 post-transcriptionally regulate enhancer of zeste homolog 2 and inhibit migration and invasion in human esophageal squamous cell carcinoma
Source: Mol Cancer. 2012 Aug 6;11:51. doi: 10.1186/1476-4598-11-51 (PMC3496689; doi:10.1186/1476-4598-11-51)
Supplement: Additional file 3 — Figure S3.The expression levels of EZH2 was significantly increased in Eca109 cells transfected with pcDNA.EZH2. [file 1476-4598-11-51-S3.pdf]

Supplement Fig 3

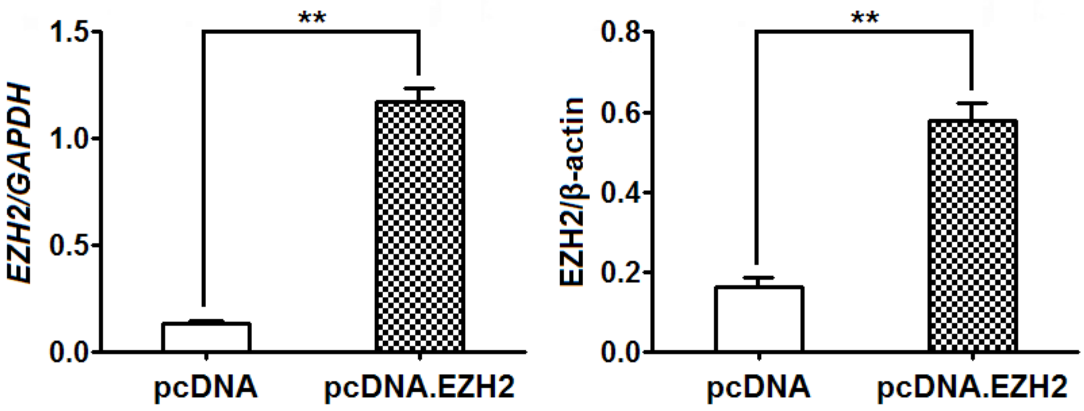

Fig.S3 The expression levels of EZH2 was significantly increased in Eca109 cells transfected with pcDNA.EZH2.

Eca109 cells were transfected with pcDNA.EZH2 (or empty pcDNA plasmid). The expression level of EZH2 mRNA and protein was detected by qRT-PCR and western blot and normalized to that of GAPDH and β-actin at 48 hr post-transfection, respectively. Histogram showed the expression level of EZH2 mRNA (A) and protein (B) in pcDNA.EZH2-transfected and empty pcDNA-transfected Eca109 cells. **, $P < 0.01$ by t test.
